# Natural α-Amylase Inhibitors from Medicinal Herbs: In Vitro Evaluation of Extracts Prepared with Food-Compatible Solvents

**DOI:** 10.3390/foods15111843

**Published:** 2026-05-23

**Authors:** Mihailo Mladenović, Milica Milutinović, Nevena Đukić, Mirjana Rajilić-Stojanović

**Affiliations:** 1 Faculty of Technology of Metallurgy, University of Belgrade, Karnegijeva 4, 11000 Belgrade, Serbia; mladenovicm@tmf.bg.ac.rs (M.M.); mmilutinovic@tmf.bg.ac.rs (M.M.); 2 Innovation Center, Faculty of Technology and Metallurgy, University of Belgrade, Karnegijeva 4, 11000 Belgrade, Serbia; ndjukic@tmf.bg.ac.rs

**Keywords:** diabetes mellitus, medicinal herbs, polyphenols, α-amylase, antioxidants, antimicrobial activity, functional foods, enzyme inhibition

## Abstract

Medicinal plants represent a promising source of bioactive compounds with potential antidiabetic activity, while the efficacy of plant extracts depends on both plant matrix and extraction conditions. This study aimed to systematically compare selected medicinal plants and extraction solvents to evaluate their impact on extracts’ in vitro α-amylase inhibitory activity, total polyphenol content (TPC), antioxidant capacity, and antimicrobial properties. Extracts of sage (*Salvia officinalis*), blueberry leaf (*Vaccinium myrtillus*), nettle (*Urtica dioica*), wormwood (*Artemisia absinthium*), and green and roasted coffee (*Coffea arabica*) were prepared using different solvent systems (50% (*v*/*v*) ethanol, propylene glycol, glycerol, and water), as well as a traditional aqueous infusion protocol. Extraction solvent strongly affected bioactivity: ethanol extracts showed the highest α-amylase inhibition, particularly in sage extract (79.60%) and blueberry leaf (57.71%). No significant correlation with TPC was observed (r = 0.229, *p* = 0.108), but aqueous ethanol yielded the highest TPC, with blueberry leaf being richest (64.16 ± 0.82 mg GAE/g), followed by roasted coffee (49.36 ± 0.83 mg GAE/g). Ethanol extracts demonstrated antibacterial activity against *Staphylococcus aureus*. Overall, sage and blueberry leaves showed the most promising multifunctional activity, highlighting their potential for further investigation as functional food ingredients.

## 1. Introduction

Medicinal and aromatic plants have been used for centuries in traditional medicine and human nutrition, providing a longstanding empirical basis for their health-related effects. They are rich in phenolic compounds with diverse biological activities, including antioxidant, anti-inflammatory, and enzyme-modulating properties [1,2]. These compounds can interact with digestive enzymes through non-covalent mechanisms such as hydrogen bonding and hydrophobic interactions, reducing catalytic activity and substrate accessibility [3]. In particular, several medicinal plants have been reported to inhibit key amylolytic enzymes involved in carbohydrate digestion, such as α-amylase and α-glucosidase [4]. Therefore, preparations based on medicinal plants can be used for controlling glycemic response to foods. In addition to enzyme inhibition, the overall functional potential of plant extracts is influenced by their polyphenol content, and inherent antioxidant activity, while their antimicrobial and prebiotic properties may further contribute to both food preservation and health-promoting effects.

The modulation of postprandial glucose levels through dietary means has become an important research focus in food science and nutrition. The rate and extent of starch digestion determine the glycemic index of foods, which directly influence metabolic health and the risk of chronic diseases such as type 2 diabetes mellitus and obesity [5]. Importantly, starch that escapes digestion in the small intestine is classified as resistant starch and can exert significant effects on the gut microbiota through colonic fermentation. Resistant starch has been associated with a range of beneficial health effects, including improved metabolic outcomes and intestinal health [6]. Strategies that delay starch hydrolysis, particularly through the inhibition of α-amylase and α-glucosidase, are therefore of great interest for developing functional foods with a low glycemic response [7]. Importantly, the benefits of such approaches are not limited to slower glucose release; delayed starch digestion increases the fraction of starch reaching the colon, making it available to the gut microbiota. This shift in substrate availability promotes microbiota modulation, which in turn contributes to improved metabolic health [8]. Importantly, pharmacological strategies targeting key digestive enzymes have also been developed to control postprandial glycemia. Enzyme inhibitors such as acarbose are effective but often cause gastrointestinal side effects, prompting increasing interest in alternative inhibitors, such as those derived from edible and medicinal plants [9]. The inhibitory potential of medicinal plant preparations depends on structural features such as hydroxylation pattern and molecular weight of extracted compounds, which are highly dependent on the extraction conditions and solvent polarity [3]. Modulating these parameters enables the design of more effective and technologically suitable plant-derived inhibitors for incorporation into functional food formulations.

Several medicinal herbs used in commercial anti-diabetic, plant-based formulations have shown promising α-amylase inhibitory potential. These formulations are typically based on traditional empirical approaches, where combinations of plants are selected without a detailed understanding of the contribution of individual components. In this study, selected plant materials were analyzed individually, including components commonly used in commercial herbal formulations as well as plants frequently cited in the literature for their hypoglycemic properties, for comparison. Green and roasted coffee beans (*Coffea arabica*) beans are rich in chlorogenic acids known to delay glucose absorption and modulate lipid metabolism [10]. Common nettle (*Urtica dioica*) provides flavonoids and phenolic acids with antioxidant and hypoglycemic properties [11]. *Vaccinium* species, including blueberry leaves, are abundant in anthocyanins and proanthocyanidins that affect carbohydrate metabolism and insulin sensitivity [12]. Common wormwood (*Artemisia absinthium*) contains sesquiterpene lactones and phenolics linked to anti-obesity and antidiabetic effects [13], while sage (*Salvia officinalis*) exhibits strong α-amylase and α-glucosidase inhibition due to its rosmarinic acid and flavone content [14].

Building on these observations, the present study investigated the α-amylase inhibitory activity of extracts obtained from green and roasted coffee beans, common nettle leaves, blueberry leaves, common wormwood leaves, and sage leaves. In order to comprehensively characterize their functional potential, total polyphenol content, antioxidant capacity, and antimicrobial activity against selected bacterial and yeast strains were also evaluated. Environmentally friendly extraction approaches with food-grade solvents, such as hydroalcoholic or glycerol-based solvents, were employed to enable efficient polyphenol recovery while ensuring their applicability in food formulations [15]. Such approaches align with global efforts to integrate green chemistry principles into food processing and bioactive compound research. The findings enable a more targeted selection of plant extracts for the development of functional formulations aimed at controlling starch digestibility and glycemic response. Although the investigated plant materials have been previously studied individually, direct comparison of their bioactivity is limited by differences in extraction conditions, solvents, analytical protocols, and data expression. In this study the standardized comparative evaluation of selected medicinal plant materials under identical experimental conditions was performed. The inclusion of the Gluko Mix herbal infusion as a reference formulation further allows comparison between individual plant materials and a commercially available herbal blend.

## 2. Materials and Methods

### 2.1. Material

Six types of plant materials were investigated in this study: finely ground green coffee beans (*Coffea arabica*), roasted coffee beans (*Coffea arabica*), common nettle leaves (*Urtica dioica* L.), blueberry leaves (*Vaccinium myrtillus*), common wormwood leaves (*Artemisia absinthium* L.), and sage leaves (*Salvia officinalis* L.). Green coffee beans, harvested in Brazil, were supplied by ‘Sucafina’, (Geneva, Switzerland), while roasted coffee beans were commercially obtained under the brand name ‘Fina kafa’ (Belgrade, Serbia). Medicinal plant materials (sage, common nettle, blueberry, and wormwood leaves) were purchased from the Institute for Medicinal Plant Research “Dr. Josif Pančić” (Belgrade, Serbia). Blueberry, wormwood, and sage leaves constitute components of the Gluko Mix herbal dietary formulation intended to support normal glucose metabolism and developed by the same institute. Gluko Mix contains bean pod (*Phaseoli legumen*, 30% (*w*/*w*)), birch leaf (*Betulae folium*, 20% (*w*/*w*)), bilberry leaf (*Myrtilli folium*, 20% (*w*/*w*)), black mulberry leaf (*Mori nigrae folium*, 10% (*w*/*w*)), olive leaf (*Olivae folium*, 10% (*w*/*w*)), wormwood herb (*Absinthii herba*, 5% (*w*/*w*)), and nettle leaf (*Urticae folium*, 5% (*w*/*w*)).

For extract preparation, the following solvents were used: ethanol (96% (*v*/*v*); Zorka Pharm, Šabac, Serbia), glycerol (99% (*v*/*v*); Zorka Pharm, Šabac, Serbia), propylene glycol (99% (*v*/*v*); Riedel–de Haën, Seelze, Germany), and distilled water.

Porcine pancreatic α-amylase (Sigma-Aldrich, St. Louis, MO, USA), sodium dihydrogen phosphate (NaH_2_PO_4_; Lach-Ner, Neratovice, Czech Republic), starch (Gramma Libero Tehnika d.o.o., Belgrade, Serbia) and 3,5-dinitrosalicylic acid (DNS) reagent were used for the determination of relative α-amylase inhibition (RI). Total polyphenol content (TPC) was determined using Folin–Ciocalteu reagent (Merck, Darmstadt, Germany), gallic acid standard (Sigma-Aldrich, USA), and sodium carbonate (Na_2_CO_3_; Lach-Ner, Czech Republic). Acarbose (Tokyo Chemical Industry Co., Ltd., Tokyo, Japan) was employed as a positive control for the α-amylase inhibition assay.

Ferric reducing antioxidant power (FRAP) was evaluated using 2,4,6-tris(2-pyridyl)-1,3,5-triazine (TPTZ, 40 mmol/L; TCI Chemicals, Tokyo, Japan), iron(III) chloride hexahydrate (FeCl_3_·6H_2_O, 20 mmol/L), sodium acetate trihydrate (CH_3_COONa·3H_2_O), glacial acetic acid (CH_3_COOH), and hydrochloric acid (HCl, 40 mmol/L).

Antimicrobial activity was assessed using the resazurin microdilution assay. Tryptone soy broth (TSB), resazurin sodium salt (Acros Organics, Geel, Belgium), and reference microbial strains *Escherichia coli* ATCC 25922, Staphylococcus aureus ATCC 25923, and *Candida albicans* ATCC 10259 were used.

### 2.2. Extract Preparation

Extracts of five selected plant materials (green or roasted coffee beans, blueberry leaves, nettle leaves, wormwood leaves, and sage leaves) were prepared using four different solvents: 50% (*v*/*v*) glycerol, 50% (*v*/*v*) propylene glycol, 50% (*v*/*v*) ethanol, and distilled water and two extraction procedures.

Protocol 1: 3.0 g of finely chopped plant material was mixed with 30 mL of the respective solvent, corresponding to a liquid-to-solid ratio of 10 mL/g. Extractions were carried out at 60 °C for 1 h under constant agitation [16]. After extraction, the mixtures were vacuum filtered, and the obtained supernatants were collected and stored in the dark at 4 °C until further analysis.

Protocol 2: aqueous extracts were prepared using boiling water to mimic the traditional preparation of the Gluko Mix herbal blend herbal infusion. This procedure was applied both to individual plant materials and to the Gluko Mix formulation, which served as a reference beverage for comparison of biological activity. Three tablespoons (equivalent to 9 g) of the plant material were infused with 800 mL of boiling water, boiled for 10 min, and subsequently vacuum filtered. The resulting supernatants were stored in the dark at 4 °C until analysis. The protocols differed in extraction time and temperature (1 h at 60 °C vs. 10 min in boiling water), solvent-to-solid ratio (10 mL/g vs. 88 mL/g). Protocol 1 included water and different solvent systems under controlled conditions to compare solvent efficiency, whereas protocol 2 used only water and was designed to simulate the traditional preparation of the Gluko Mix herbal infusion.

### 2.3. Total Polyphenol Content Determination (TPC)

The total polyphenol content (TPC) of plant extracts was quantified using the Folin–Ciocalteu colorimetric method as previously described [17]. Briefly, 50 µL of the extract was combined with 250 µL of Folin–Ciocalteu reagent and 3.7 mL of distilled water. After thorough mixing, 1.0 mL of 20% sodium carbonate solution was added to initiate the reaction. The mixture was then incubated for 2 h at room temperature in the dark to ensure complete color development. Absorbance was recorded at 750 nm against a reagent blank. Gallic acid served as the calibration standard in concentrations ranging from 0.02 to 1.5 mg/mL. The results were calculated from the standard curve (y = 0.8488 × x) and expressed as gallic acid equivalents (GAE) per gram of plant material.

### 2.4. Ferric Ion Reducing Antioxidant Power (FRAP)

The antioxidant capacity of plant extracts was evaluated using the ferric reducing antioxidant power (FRAP) assay, which relies on the reduction of the Fe^3+^–TPTZ (iron(III)–tripyridyltriazine) complex to its ferrous form (Fe^2+^–TPTZ), producing a blue chromophore under acidic conditions [18]. The FRAP working solution was freshly prepared by combining 0.3 M acetate buffer (pH 3.6), 10 mM TPTZ dissolved in 40 mM HCl, and 20 mM FeCl_3_·6H_2_O in a volumetric ratio of 10:1:1. To perform the assay, 50 µL of the extract was mixed with 1.5 mL of FRAP reagent, vortexed, and incubated for 5 min in the dark at room temperature. The absorbance of the resulting solution was then recorded at 593 nm against a reagent blank. A calibration curve was constructed using FeSO_4_·7H_2_O standards in the range of 0.2–1.0 mmol/L, and the results were expressed as millimoles of Fe^2+^ equivalents per gram of plant material.

### 2.5. Determination of the Relative Inhibitory Effect on α-Amylase Activity (RI)

The relative inhibitory (RI) activity of plant extracts against α-amylase was assessed using the 3,5-dinitrosalicylic acid (DNS) assay, which quantifies reducing sugars generated as a result of α-amylase-mediated hydrolysis [19]. This method is based on the reduction of DNS by reducing sugars to a coloured reduced product, with absorbance measured at 540 nm. The reaction mixture contained 20 µL of plant extract, 20 µL of α-amylase solution (2.5 mg/mL; final enzyme activity in the assay: 0.7 IU)), and 160 µL of sodium phosphate buffer (pH 6.9). After pre-incubation for 5 min at 37 °C, the reaction was initiated by adding 200 µL of a 1% starch solution and incubated for another 5 min at the same temperature. The enzymatic reaction was then terminated by adding 400 µL of DNS reagent, followed by heating in a boiling water bath for 5 min to develop the colour. After cooling, each mixture was diluted with 3 mL of distilled water, and absorbance (*A*_1_) was recorded at 540 nm against a blank. Control samples without enzyme and with plant extract were included to correct for non-enzymatic starch degradation and potential interference with reduction of DNS reagent. Enzyme-only controls (without extract) represented 100% enzymatic activity (*A*_0_). Inhibitory activity of the corresponding extraction solvents (50% (*v*/*v*) ethanol, propylene glycol, and glycerol) was determined under the same assay conditions. The percentage inhibition of α-amylase was calculated by comparing the reducing sugar content in the presence and absence of the plant extract (1):

(1) $$Relative\;inhibition\left(\%\right)=\frac{\left(A_{0}-A_{1}\right)}{A_{0}}*100$$

$$A_{0}—enzyme\text{-}only\;control\;apsorbance,$$

$$A_{1}—sample\;absorbance.$$

For the two ethanolic extracts exhibiting the highest α-amylase inhibitory activity (sage and blueberry leaves), as well as for acarbose as the reference inhibitor, IC_50_ values were determined using the same assay procedure across a range of extract and compound concentrations.

### 2.6. Assessment of Antimicrobial Activity by Resazurin Assay

Resazurin (7-hydroxy-3H-phenoxazin-3-one 10-oxide) is a phenoxazine-based dye that is weakly fluorescent, non-toxic, cell-permeable, and sensitive to redox reactions. The dye exhibits a purple colour at pH values above 6.5, while it appears orange at pH values below 3.8. Owing to its redox sensitivity, resazurin is widely applied in microbiological, cellular, and enzymatic assays, as it can be irreversibly reduced to resorufin by metabolically active cells as a result of cellular respiration. Resorufin is a pink compound with strong fluorescence, enabling the detection of metabolic activity through a color change in the assay solution.

The assay was performed in 96-well microplates by combining 100 μL of tryptic soy broth (TSB), 100 μL of the tested sample (20 mg/mL), 10 μL of microbial suspension (final concentration 5 × 10^6^ CFU/mL), and 10 μL of resazurin solution. For each tested sample, a corresponding control without the microbial inoculum was included, containing all components except the microbial suspension. In addition, a microbial growth control was prepared containing all components except the tested plant extract [20]. The corresponding extraction solvents (50% (*v*/*v*) ethanol, 50% (*v*/*v*) propylene glycol, and 50% (*v*/*v*) glycerol) were also tested as solvent controls under the same experimental conditions, while vancomycin (stock concentration 1 mg/mL) was used as a positive antibiotic control. The resazurin stock solution was prepared by dissolving 270 mg of resazurin in 40 mL of distilled water, followed by sterile filtration through a 0.45 μm membrane filter. All results are expressed as minimal inhibitory concentrations—with MIC defined as the lowest concentration of plant material (mg initial plant material/mL) that prevented resazurin reduction, as indicated by the absence of colour change.

### 2.7. Statistical Analysis

Data are expressed as mean ± standard deviation (SD) of two independent experiments performed from the extraction step onward. All mentioned results were subjected to two-way (ANOVA) analysis. *p*-value ≤ 0.05 was considered to be statistically significant. The Pearson test was also applied in order to determine the correlation between the results.

During the preparation of this manuscript, the authors used ChatGPT (OpenAI, GPT-5.3) for the purposes of formatting text only.

## 3. Results

### 3.1. α-Amylase Inhibitory Activity of Plant Extracts

**Figure 1:**
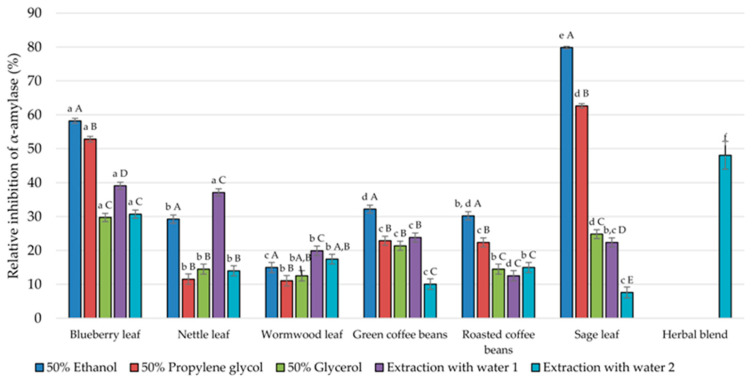
Relative α-amylase inhibition (%) of plant extracts obtained using different extraction systems. Values are presented as mean ± SD (n = 2). Different lowercase letters (a–f) above the bars indicate significant differences among plant materials within the same extraction system (*p* < 0.05), while different uppercase letters (A–E) indicate significant differences among extraction solvents/procedures for the same plant material.

The α-amylase inhibitory activity of plant extracts was evaluated comparatively to assess the effects of both plant source and extraction solvent on their antidiabetic potential. The relative inhibition (RI) of α-amylase was determined for extracts obtained using different solvents. Considerable variability in inhibitory activity was observed among the investigated plant materials and extraction systems. Among the tested solvents, ethanol extracts generally exhibited the strongest inhibitory potential. Within this group the strongest inhibition of α-amylase was observed for ethanol extracts of sage leaf (79.60%), followed by blueberry leaves (57.71%), whereas wormwood leaf showed the lowest inhibitory activity (16.02%, Figure 1).

A similar pattern was observed for propylene glycol extracts, although overall inhibitory activity was slightly lower compared to ethanol extracts. Sage leaf again showed the highest RI value (62.65%), followed by blueberry leaf (52.82%). In contrast, extracts of green coffee beans and roasted coffee beans exhibited similar and notably lower inhibitory activity (22.84% and 22.35%, respectively). Nettle and wormwood extracts displayed the lowest RI values.

Glycerol extracts demonstrated substantially lower inhibitory activity across all investigated plant materials. The highest inhibition within this solvent system was observed for blueberry leaf extract (29.72%), followed by sage leaf (24.80%) and green coffee bean extract (21.36%). Roasted coffee bean extract and nettle leaf extract showed similar inhibition values (14.48%), whereas wormwood leaf extract exhibited the lowest activity (12.52%).

For aqueous extracts obtained using water extraction protocol 1, the strongest inhibition was observed for blueberry leaf (39.06%) and nettle leaf (37.09%). Moderate inhibition was detected for green coffee beans (23.82%) and sage leaf (22.35%), whereas wormwood (19.89%) and roasted coffee bean extracts (12.52%) exhibited lower activity. In contrast, aqueous extracts prepared using water extraction protocol 2 generally displayed lower α-amylase inhibitory activity. Blueberry leaf extract again showed the highest RI value (30.70%), followed by wormwood (17.43%) and roasted coffee bean extract (14.97%). Nettle leaf extract showed similar inhibition (13.99%), while green coffee bean (10.06%) and sage leaf extracts (7.60%) demonstrated the weakest activity among the tested samples. The herbal blend extract prepared using water protocol 2 exhibited relatively high inhibitory activity (48.06%), exceeding the RI values of the individual plant extracts obtained using the same extraction procedure.

The inhibitory activity of the extraction solvents alone was 7.2% for ethanol, 7.4% for propylene glycol, and 8.04% for glycerol, with no statistically significant differences among the tested solvents. Since the ethanol extracts of sage and blueberry leaf demonstrated the highest α-amylase inhibitory activity among all tested samples, their inhibitory potency was further characterized by determining IC_50_ values. Sage ethanol extract exhibited a lower IC_50_ value (618 ± 9 µg/mL) than blueberry leaf ethanol extract (770 ± 21 µg/mL), indicating stronger α-amylase inhibitory potency of sage. Acarbose, used as a reference inhibitor, exhibited a significantly lower IC_50_ value of 2.76 ± 0.02 µg/mL under the same experimental conditions.

### 3.2. Total Polyphenol Content and Antioxidant Capacity of Plant Extracts

To complement the evaluation of enzyme inhibitory activity, total polyphenol content (TPC) and antioxidant capacity (FRAP) were determined for extracts prepared from blueberry leaf, nettle leaf, wormwood leaf, green and roasted coffee beans, sage leaf, and the ‘Gluko Mix’ herbal blend formulation. Extractions were performed using ethanol, propylene glycol, glycerol, and water. Marked variability in bioactivity was observed among the investigated plant materials across all solvent systems (Table 1 and Table 2). The highest TPC in the extracts was recorded for blueberry leaf water extract (70.25 mg GAE/g), while nettle leaf water extract exhibited the lowest value (9.26 mg GAE/g). Correspondingly FRAP values ranged from 0.06 to 0.56 mmol Fe^2+^/g. A strong and statistically significant correlation between TPC and FRAP values was observed (r = 0.936, *p* < 0.001). In contrast to antioxidant capacity, α-amylase inhibition was not significantly correlated with TPC (r = 0.229, *p* = 0.108).

Two extraction approaches were used: controlled extraction with 50% (*v*/*v*) ethanol, propylene glycol, and glycerol, alongside water, to compare solvent efficiency, and boiling water extraction to simulate traditional herbal infusion preparation.

The extraction solvent markedly influenced both the recovery of phenolic compounds and the antioxidant capacity of the investigated extracts. Overall, hydroalcoholic extraction with 50% (*v*/*v*) ethanol yielded the highest TPC and reducing power, whereas glycerol extracts generally exhibited the lowest antioxidant capacity. Propylene glycol extracts showed intermediate behavior, indicating that solvent polarity and extraction conditions strongly affect the efficiency of phenolic compound recovery from plant matrices. The second aqueous extraction protocol also resulted in relatively high total polyphenol contents and reducing power compared to most solvent extracts, which may be related to the higher extraction temperature and larger solvent-to-solid ratio applied in this protocol.

A comparison among individual plant materials further highlights these differences. Blueberry leaf extracts consistently exhibited the highest reducing capacity across most solvent systems. For example, when extracted with 50% (*v*/*v*) ethanol, the reducing power of blueberry leaf extract was more than five times higher than that of nettle leaf extracted under the same conditions. A similar trend was observed for propylene glycol extracts, where blueberry extracts showed approximately threefold higher FRAP values compared with wormwood extracts. Coffee extracts also displayed notable antioxidant capacity; roasted coffee beans consistently exhibited higher polyphenol levels than green coffee beans in several solvent systems, although this difference did not always correspond to proportionally higher reducing power. These observations indicate that both the botanical source and the extraction solvent play a crucial role in determining the phenolic composition and antioxidant potential of the obtained extracts, and further suggest that the qualitative profile of phenolic compounds may be more important than their total concentration alone.

A two-way ANOVA was performed to evaluate the effects of plant material and extraction solvent on total polyphenol content (TPC), antioxidant capacity (FRAP), and α-amylase inhibitory activity (RI). A significant interaction between plant material and solvent was observed and evaluation of functional properties confirmed that the effect of extraction conditions depended on the specific plant matrix, with some solvents enabling clearer differentiation among plant extracts than others.

### 3.3. Antimicrobial Activity of Selected Plant Extracts

**Figure 2:**
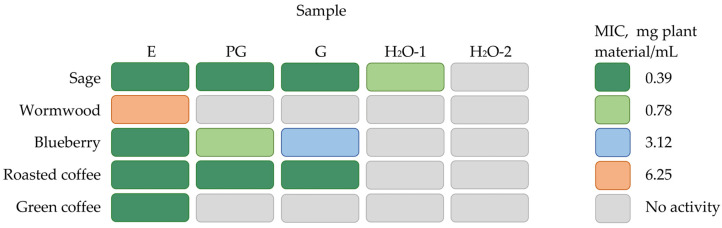
Minimal inhibitory concentrations (MIC, mg/mL) of selected plant extracts prepared in different solvents against *S. aureus*. Abbreviations: E, 50% (*v*/*v*) ethanol; PG, 50% (*v*/*v*) propylene glycol; G, 50% (*v*/*v*) glycerol; H_2_O-1, water extract obtained by Protocol 1; H_2_O-2, water extract obtained by Protocol 2.

The antimicrobial potential of the investigated plant extracts was assessed by determining minimal inhibitory concentrations (MIC, mg/mL) against *Escherichia coli*, *Staphylococcus aureus*, and *Candida albicans.* Inhibitory activity was detected only against *S. aureus* (Figure 2), whereas no growth inhibition was observed against *E. coli* or *C. albicans* under the tested conditions.

Among the evaluated samples, sage leaf extracts exhibited notable antibacterial activity against *S. aureus*, with MIC values of 0.39 mg/mL for the ethanol, propylene glycol, and glycerol extracts, while the aqueous extract showed weaker inhibition (MIC 0.78 mg/mL). Blueberry leaf extracts also demonstrated solvent-dependent activity against *S. aureus*, with the strongest effect observed in the ethanol extract (MIC 0.39 mg/mL), followed by propylene glycol (MIC 0.78 mg/mL) and glycerol (MIC 3.13 mg/mL). Extracts of roasted coffee beans showed consistent inhibition of *S. aureus*, with MIC values of 0.39 mg/mL for ethanol, propylene glycol, and glycerol extracts. In contrast, green coffee beans exhibited antibacterial activity only in the ethanol extract (MIC 0.39 mg/mL), while the remaining solvent systems showed no measurable inhibition. Wormwood leaf extracts displayed limited antimicrobial efficacy, with inhibition detected only for the ethanol extract at a substantially higher concentration (MIC 6.25 mg/mL). Nettle leaf extracts did not exhibit detectable antimicrobial activity, as no MIC values were recorded for any microorganism or solvent condition. Finally, no MIC values were recorded for extracts obtained using water extraction procedure 2, as no detectable antimicrobial activity was observed against the tested microorganisms under the applied experimental conditions. MIC was also determined for solvent systems. Against *S. aureus*, ethanol and propylene glycol exhibited MIC values of 12.5% (*v*/*v*), while glycerol showed weaker activity with an MIC of 25% (*v*/*v*). Therefore, solvent concentration present at the inhibitory level of the extracts was considerably lower than the MIC of solvents. For instance, an extract with an MIC of 6.25 mg/mL contained 3.13% (*v*/*v*) solvent, which is well below the MIC of solvent alone. Additionally, vancomycin was included as a reference antibiotic control, and its MIC against *S. aureus* was determined to be 62.5 μg/mL.

## 4. Discussion

Medicinal plants represent an underexplored source of bioactive phytochemicals with significant potential for application in functional foods and nutraceuticals. In particular, plant polyphenols have attracted considerable attention due to their antioxidant, antimicrobial, and enzyme-inhibitory activities. These bioactive properties are often harnessed not only through individual plant extracts but also through combinations of multiple plant species. Polyherbal formulations are widely used in traditional herbal medicine and functional beverages, where mixtures of several plant species are often assumed to provide enhanced or synergistic biological effects. In many cases, herbal blends are formulated based on empirical knowledge or traditional practice rather than systematic experimental validation.

An additional important aspect relates to the mode of preparation. Herbal infusions prepared in water represent the most traditional approach, consistent with procedures commonly recommended by the European Medicines Agency for many medicinal plants [21]. However, extraction efficiency may be improved by the use of alternative food-grade solvents [22]. Furthermore, complex plant mixtures prepared as infusions may exhibit sensory properties that reduce consumer acceptability [23]. In this context, extracts obtained using alternative solvents may offer a more practical strategy for delivering bioactive compounds, for example, in the form of concentrated liquid extracts or encapsulated formulations [24].

Accordingly, the present study aimed to evaluate the biological activity of individual plant components of a representative herbal blend intended for glucose control. In parallel, several food-compatible solvents were tested under standardized extraction conditions to compare their efficiency in recovering bioactive compounds from individual plant materials. The performance of these extracts was then compared with that of the herbal blend prepared according to a traditional aqueous extraction protocol (boiling water), reflecting its typical mode of use. This approach enabled a systematic screening of both plant matrices and extraction conditions in order to identify the most promising candidates for further development. Sage (*Salvia officinalis*) and coffee (*Coffea arabica*) were additionally included as a reference plant due to its well-documented use in glycemic control and digestive health, as supported by several review studies [25,26,27,28].

In the present study the investigated plant extracts exhibited considerable variation in α-amylase inhibitory activity, although several demonstrated notable effects, supporting their potential relevance in dietary strategies aimed at controlling postprandial glycemia. Sage extracts showed particularly strong inhibitory effects, which is consistent with previous reports describing *Salvia officinalis* as a promising source of natural carbohydrate-digesting enzyme inhibitors [29]. Interestingly, blueberry leaf extracts exhibited comparable and substantial inhibitory activity, suggesting that this plant material represents an underexplored source of bioactives. Research on *Vaccinium* species has traditionally focused on the fruit, whose polyphenol-rich extracts have been reported to inhibit amylolytic enzymes and modulate carbohydrate digestion [30]. While the biological activity of blueberry leaves has also been reported [31], the present study places these observations in a broader comparative context by directly evaluating blueberry leaves alongside several other medicinal plants under standardized extraction conditions, highlighting their strong α-amylase inhibitory potential.

Compared to individual plant extracts, the herbal blend exhibited intermediate biological activity, indicating that combining multiple plant materials does not necessarily result in enhanced effects. The herbal blend showed a relative inhibition of α-amylase of 45.16%, which was lower than that observed for ethanol extracts of sage (79.60%) and blueberry leaf (57.71%), but higher than the inhibition recorded for wormwood (16.02%). A similar trend was observed for total polyphenol content and antioxidant capacity, which were moderate relative to individual plant matrices (TPC 29.79 mg GAE/g; FRAP 0.066 mmol Fe^2+^/g). Furthermore, combining multiple plant materials did not improve the measured bioactivities compared to selected individual extracts. From a functional perspective, these findings indicate predominantly additive rather than synergistic effects within the tested herbal blend.

Importantly, although the herbal blend was prepared using an aqueous extraction protocol with a high solvent-to-solid ratio, its polyphenol yield and biological activity remained lower than those obtained with hydroalcoholic solvents. This suggests that extraction efficiency is governed primarily by solvent polarity and its ability to solubilize specific classes of phenolic compounds. It should be noted that the baseline inhibitory activity of the pure extraction solvents alone was notably lower than that of the plant extracts, and similar among all tested solvents. These findings indicate that α-amylase inhibitory activity observed for the ethanol plant extracts is primarily attributable to the enriched presence of extracted bioactive compounds rather than the solvent vehicle itself. Nevertheless, the minor, measurable background effects of the pure solvents suggest that this contribution should be carefully considered when interpreting the final net activity of the extracts. In addition, it should be noted that the α-amylase inhibition values were compared under standardized extraction and assay conditions; however, they were not normalized to extraction yield or dry extract mass. Therefore, cross-solvent comparisons should not be interpreted as direct measures of intrinsic inhibitory potency.

Ethanol-based systems are known to enhance the recovery of a broader range of phenolics, particularly less polar flavonoids, which may contribute more significantly to enzyme inhibition [32]. In contrast, glycerol extracts showed weaker enzyme inhibition despite moderate phenolic levels, suggesting that solvent polarity influences the extraction of specific bioactive constituents. This is consistent with previous studies demonstrating the suitability of hydroalcoholic solvents for phenolic compound extraction [33]. In the present study, solvent recovery, energy demand, extraction yield, downstream concentration and drying steps were not evaluated. Therefore, statements regarding the practical applicability should be interpreted as a basis for further process optimization.

The strongest inhibition of α-amylase in this study was observed for ethanol extracts of sage leaves. The high intrinsic potential of *S. officinalis* is documented in the literature in studies that used organic solvents in extraction. For instance, research utilizing dichloromethane as a solvent reported an IC_50_ value of 71.20 μg/mL [34], while an even lower value of 46.52 μg/mL was obtained for an ethyl acetate fraction of methanol sage extract [14]. Our ethanol extract achieved an IC_50_ of 618 μg/mL indicating a lower potency. However, it should be noted that a direct comparison between different assay systems is limited by variations in enzyme concentration and incubation parameters.

The significance of our result becomes apparent given contextualized biological activity within the constraints of food-safe extractions. It is well established that food-grade solvents, such as water or aqueous ethanol, typically yield extracts with lower biological activity compared to aggressive organic systems. For instance, the literature reports for the *Salvia* genus frequently cite IC_50_ values in the milligram range, for example, IC_50_ of 19.08 mg/mL for *S. virgata* alcoholic extracts [35], and 4.9 ± 0.4 and 10.3 ± 0.9 mg/mL for aqueous extracts of *S. judaica* and *S. multicaulis*, respectively [36]. It should be emphasized that IC_50_ values are strictly comparable only when determined within the same experimental system. When evaluated under internally consistent conditions, a stark dichotomy emerges: extracts obtained with aggressive or toxic organic solvents often exhibit IC_50_ values in the same order of magnitude as acarbose [14,34]. Notably, our ethanol extracts of *S. officinalis* and *V. myrtillus* leaves exhibited a difference in inhibitory potency relative to acarbose of approximately 220-fold for *S. officinalis* and 280-fold for *V. myrtillus*. This represents a substantial improvement compared to many conventional food-grade extracts reported in the literature, for which IC_50_ values can be up to 20,000-fold higher than that of the pharmaceutical standard [36]. Furthermore, these two plants demonstrated significantly higher α-amylase inhibition than coffee extracts, despite coffee being a well-established dietary source of antidiabetic compounds [26]. In previous research, the IC_50_ of *V. myrtillus* leaf extracts could not be determined within the tested range [31]. Notably, previous research failed to determine an IC_50_ for *V. myrtillus* leaf extracts even at concentrations up to 1000 μg/mL [31]. While that study utilized a similar hydro-ethanolic solvent as we did, the extraction was conducted overnight without heating. The fact that our thermal extraction yielded a potent IC_50_ (633 μg/mL) despite a shorter duration suggests that the recovery of inhibitors is critically dependent on extraction temperature. This suggests that α-amylase inhibition is heavily dependent on the specific chemical profile yielded by the extraction process, where temperature and solvent play a critical role.

In line with this notation, it is important to stress that aqueous infusions, although traditional and practical, may not provide optimal recovery of bioactive compounds. The observed reduced activity of the herbal infusion may be partially attributed to thermal degradation of phenolic compounds during boiling. Elevated temperatures and prolonged exposure to heat have been reported to decrease the stability of flavonoids and phenolic acids [32,37]. It should be noted, however, that these conclusions are based on a limited set of in vitro parameters and may not fully reflect the complexity of interactions occurring in vivo, where the overall effect of a herbal blend extract depends on factors such as digestion, absorption, metabolism, and interactions with the gut microbiota. Nevertheless, obtained observations underscore the importance of experimental validation in the design of herbal formulations.

The strong correlation observed between TPC and FRAP values further supports the widely reported role of polyphenolic compounds as major contributors to the antioxidant activity of plant extracts [38]. Although polyphenols are frequently associated with α-amylase inhibition, the results of the present study indicate that TPC alone does not fully explain the observed inhibitory activity. For example, roasted coffee bean extracts contained relatively high levels of polyphenols but showed comparatively low α-amylase inhibition. Similar discrepancies have been reported in previous studies, suggesting that enzyme inhibition depends more strongly on the structure and specific composition of phenolic compounds than on their total concentration [39,40]. Polyphenols interact with digestive enzymes primarily through non-covalent interactions, including hydrogen bonding and hydrophobic interactions, which may alter enzyme conformation and reduce catalytic activity. The strength of these interactions depends on structural features of phenolic compounds, including the number of hydroxyl groups and molecular size. Therefore, the qualitative profile of phenolic compounds present in plant extracts may play a more important role in determining α-amylase inhibitory activity than their total abundance [3].

Coffee is widely recognized for its potential antidiabetic effects, and regular coffee consumption has been consistently associated with a reduced risk of type 2 diabetes in epidemiological studies [26]. Accordingly, coffee and coffee-derived extracts are frequently included in functional formulations targeting glycemic control [41]. However, in the present study, coffee extracts showed only moderate α-amylase inhibitory activity compared with the most active plant matrices, particularly sage and blueberry leaves. Both green and roasted coffee bean extracts exhibited measurable inhibition of the enzyme, together with relatively high total polyphenol content and antioxidant capacity. These findings are consistent with the well-documented richness of coffee in phenolic compounds, particularly chlorogenic acids [42], which are known to influence carbohydrate metabolism and may contribute to the modulation of postprandial glucose levels [43]. Interestingly, roasted coffee extracts showed somewhat higher antioxidant capacity, while the enzyme inhibitory activity remained moderate, suggesting that different classes of compounds may be responsible for the observed biological effects. Similarly, several bioactive phenolic compounds of sage and bilberry have been previously associated with antidiabetic properties. Rosmarinic acid, abundant in sage, has been reported to inhibit carbohydrate-hydrolyzing enzymes such as α-amylase and α-glucosidase through interactions with enzyme active sites and modulation of substrate accessibility [27,44]. The inhibition of ethanol bilberry fruit extracts was attributed to the presence of phenolic compounds such as chlorogenic acid, quercetin glycosides and procyanidins [30]. Antidiabetic activity of medicinal and dietary plants, such as coffee, is mediated by modulation of glucose absorption, improvement of insulin sensitivity, and the inhibitory effects of polyphenols on digestive enzymes involved in carbohydrate digestion [25,45]. It should be noted that metabolic effects in vivo likely arise from multiple complementary mechanisms rather than enzyme inhibition alone, and therefore, although our results demonstrate a higher inhibitory potency for sage and bilberry, this does not necessarily translate to an overall clinical superiority over coffee.

In addition to direct biochemical effects, it is increasingly recognized that plant extracts may influence host metabolism through modulation of the gut microbiota, which plays a key role in metabolic health [46]. One of the primary mechanisms by which the investigated extracts may exert such effects is through the modulation of carbohydrate digestion. Specifically, α-amylase inhibition can lead to an increased fraction of undigested starch reaching the colon, where it becomes available for microbial fermentation. This results in a higher production of short-chain fatty acids and the formation of resistant starch fractions, both of which are associated with beneficial effects on host energy metabolism and glycemic regulation [47]. Therefore, the observed α-amylase inhibitory activity may exert a dual beneficial effect, combining direct modulation of starch digestion with indirect microbiota-mediated metabolic benefits due to change in substrate availability.

In parallel, the antimicrobial activity observed in this study suggests an additional mechanism involving direct modulation of microbial populations. Although the tested microorganisms (*Staphylococcus aureus*, *Escherichia coli*, and *Candida albicans*) represent simplified model systems, the selective antibacterial activity indicates that plant-derived compounds can differentially affect microbial growth. Such selective pressure may translate, in a more complex gut environment, into shifts in microbial community structure [48]. Among the tested samples, sage, roasted coffee, and blueberry extracts exhibited the strongest antibacterial activity against *S. aureus*, which is consistent with previous reports describing antimicrobial properties of these plant matrices [49]. Since the active extracts contained only low residual ethanol concentrations, the observed antibacterial effects were attributed primarily to plant-derived bioactive compounds, although a minor contribution of ethanol cannot be excluded. The lack of activity against *E. coli* is likely due to the intrinsic resistance of Gram-negative bacteria, whose outer membrane limits the penetration of many phenolic compounds [50]. These findings indicate that the investigated plant extracts exert selective effects on microbial growth, which may be relevant for microbiota modulation. Previous studies have shown that polyphenol-rich plant extracts can alter both the composition and metabolic activity of the gut microbiota [51,52]. Since microbiota composition and function are closely linked to metabolic health [53], further studies investigating the effects of the examined extracts on complex microbial communities would be of considerable interest.

From a broader perspective, these findings align with the traditional use of many of the investigated plants in the management of metabolic and digestive disorders. Species such as *Salvia officinalis*, *Vaccinium* leaves, and coffee-derived preparations have long been employed in herbal medicine for glycemic regulation and gastrointestinal support. A key strength of the present study lies in the direct comparison of different plant matrices and extraction solvents under controlled conditions, which allows their biological activities to be evaluated within a consistent and comparable framework. Such an approach provides clearer insight into the relative contribution of plant species and extraction conditions, which is often lacking in studies focusing on single extracts.

These findings emphasize that both plant material and extraction conditions must be carefully optimized depending on the targeted biological effect. Based on the obtained results, ethanol extracts of sage and blueberry leaves demonstrated the most pronounced α-amylase inhibitory activity, while aqueous extracts, particularly those obtained using the second protocol, yielded the highest total polyphenol content and antioxidant capacity. The lack of correlation between TPC and enzyme inhibition further indicates that biological activity is governed by specific phytochemical composition rather than total phenolic content alone.

Despite these promising results, it should be noted that the present study was designed as a comparative screening approach aimed at identifying the most promising plant materials and extraction conditions under standardized experimental settings. The experiments were conducted in vitro, which does not fully reflect the complexity of physiological conditions in the human gastrointestinal tract. Although the obtained in vitro results provide valuable preliminary insight into the biological potential of the investigated extracts, such models cannot fully replicate the complexity of physiological conditions in living organisms. Factors such as digestion, absorption, metabolism, bioavailability, and interactions with the gastrointestinal microbiota may significantly influence the actual biological effects observed in vivo. Therefore, additional in vivo studies are necessary to confirm the efficacy, safety, and physiological relevance of the observed antioxidant and enzyme inhibitory activities. In addition, the study did not include detailed characterization of individual bioactive compounds responsible for the observed effects, nor did it assess potential synergistic or antagonistic interactions within the herbal blend. Future research should therefore focus on compound-level identification, particularly in bilberry leaf and sage extracts, as the most promising candidates. Furthermore, mechanistic studies and in vivo validation, as well as evaluating the impact of these extracts on gut microbiota composition and metabolic outcomes, would provide valuable insights into the modes of action of medicinal herb bioactives.

## 5. Conclusions

The present study provides a systematic comparison of selected medicinal plants and extraction solvents under controlled conditions to evaluate their antidiabetic potential through α-amylase inhibition. The results demonstrate that both plant matrix and extraction conditions significantly influence biological activity. Ethanol (50% (*v*/*v*)) proved to be the most effective solvent for obtaining extracts with strong α-amylase inhibitory activity, particularly for sage and blueberry leaves, while aqueous extracts obtained using the second protocol showed the highest total polyphenol content and antioxidant capacity due to more intensive extraction conditions rather than solvent type alone.

Among the investigated materials, *Salvia officinalis* and *Vaccinium* leaves exhibited the most promising overall biological activity, combining enzyme inhibition, antioxidant potential, and selective antibacterial effects. These findings highlight their potential as promising candidates for further investigation in the development of functional food ingredients associated with the modulation of carbohydrate digestion and gut microbiota composition. Further studies should focus on identifying the key bioactive compounds and confirming their physiological relevance in vivo.

## Figures and Tables

**Table 1:** Total polyphenol content (TPC) of plant extracts obtained using different extraction solvents and procedures.

TPC, mg GAE/g					
	Solvents				
Plant material	50% % (*v*/*v*) Ethanol	50% % (*v*/*v*) Propylene glycol	50% (*v*/*v*) Glycerol	Water	Water
	Protocol 1				Protocol 2
Blueberry leaf	64.16 ± 0.82 ^a^^A^	44.74 ± 0.29 ^aB^	31.63 ± 0.76 ^aC^	40.20 ± 0.52 ^aD^	70.25 ± 1.87 ^aE^
Nettle leaf	12.83 ± 0.38 ^bA^	14.59 ± 0.06 ^bB^	15.72 ± 0.64 ^bC^	9.26 ± 0.32 ^bD^	20.21 ± 0.47 ^bE^
Wormwood leaf	16.53 ± 0.13 ^cA^	13.32 ± 0.19 ^cB^	11.74 ± 0.26 ^cC^	11.65 ± 0.13 ^cC^	19.55 ± 0.47 ^bD^
Green coffee beans	20.70 ± 0.12 ^dA^	20.49 ± 0.06 ^d^A	19.92 ± 0.17 ^dA^	28.70 ± 0.58 ^dB^	50.03 ± 3.28 ^cC^
Roasted coffee beans	49.36 ± 0.83 ^eA^	47.15 ± 0.51 ^eA^	42.86 ± 0.57 ^eB^	38.66 ± 0.64 ^aC^	63.62 ± 1.87 ^dD^
Sage leaf	42.95 ± 0.70 ^eA^	32.29 ± 0.32 ^fB^	32.47 ± 0.45 ^fB^	17.75 ± 0.83 ^eC^	50.37 ± 0.00 ^cD^
Herbal blend					29.79 ± 1.55 ^e^

Values are presented as mean ± SD (n = 2). For each parameter separately, different lowercase letters (a–f) within the same column indicate significant differences among plant materials within the same extraction system (*p* < 0.05), while different uppercase letters (A–E) within the same row indicate significant differences among extraction solvents/procedures for the same plant material. GAE—Gallic acid equivalents.

**Table 2:** Ferric reducing antioxidant power (FRAP) of plant extracts obtained using different extraction solvents and procedures.

FRAP, mmol Fe^2+^/g					
	Solvents				
Plant material	50% (*v*/*v*) Ethanol	50% (*v*/*v*) Propylene glycol	50% (*v*/*v*) Glycerol	Water	Water
	Protocol 1				Protocol 2
Blueberry leaf	0.424 ± 0.001 ^aA^	0.358 ± 0.013 ^aB^	0.207 ± 0.053 ^acC^	0.330 ± 0.009 ^aB^	0.561 ± 0.000 ^aD^
Nettle leaf	0.077 ± 0.001 ^bA^	0.100 ± 0.003 ^bB^	0.076 ± 0.008 ^bA^	0.056 ± 0.001 ^bC^	0.157 ± 0.013 ^bD^
Wormwood leaf	0.092 ± 0.000 ^cA^	0.077 ± 0.002 ^cB^	0.061 ± 0.000 ^bC^	0.069 ± 0.003 ^cC^	0.157 ± 0.006 ^bD^
Green coffee beans	0.232 ± 0.007 ^dA^	0.221 ± 0.000 ^dA^	0.189 ± 0.051 ^cA^	0.290 ± 0.003 ^dB^	0.379 ± 0.001 ^cC^
Roasted coffee beans	0.281 ± 0.008 ^eA^	0.273 ± 0.001 ^eA^	0.275 ± 0.000 ^aA^	0.258 ± 0.000 ^eA^	0.401 ± 0.025 ^cB^
Sage leaf	0.281 ± 0.004 ^eA^	0.245 ± 0.002 ^fAB^	0.226 ± 0.003 ^acB^	0.135 ± 0.002 ^fC^	0.372 ± 0.056 ^cD^
Herbal blend					0.066 ± 0.014 ^d^

Values are presented as mean ± SD (n = 2). For each parameter separately, different lowercase letters (a–f) within the same column indicate significant differences among plant materials within the same extraction system (*p* < 0.05), while different letters (A–D) within the same row indicate significant differences among extraction solvents/procedures for the same plant material.

## Data Availability

The original contributions presented in this study are included in the article. Further inquiries can be directed to the corresponding author.
